# Long non-coding RNA SNHG3 accelerates progression in glioma by modulating miR-384/HDGF axis

**DOI:** 10.1515/biol-2020-0066

**Published:** 2020-09-05

**Authors:** Xiaofeng Zhang, Weixin Zheng, Wenting Jiang, Ruisheng Lin, Chunyang Xing

**Affiliations:** Department of Neurosurgery, Zhangzhou Affiliated Hospital of Fujian Medical University, Zhangzhou, Fujian, China; Department of Ultrasound, Zhangzhou Affiliated Hospital of Fujian Medical University, No. 59 Shengli Road, Zhangzhou, Fujian, China

**Keywords:** SNHG3, miR-384, HDGF, glioma, progression

## Abstract

Glioma is a malignant primary brain tumor that occurs in the central nervous system and has threatened the well-being of millions of patients. It is well acknowledged that long non-coding RNA (lncRNA) SNHG3 participates in the regulation of proliferation, inflation, differentiation, and metastasis in many cancers. However, the regulatory effect of SNHG3 on glioma progression is still controversial. The expression of SNHG3 and HDGF was upregulated, whereas miR-384 was downregulated in glioma tissues, compared with the normal tissues. Interestingly, high SNHG3 contributed to low survival rate while low SNHG3 showed the opposite result. Moreover, SNHG3 or HDGF knockdown significantly suppressed proliferation, migration, and invasion and induced apoptosis in glioma. Meanwhile, restoration of HDGF abrogated the inhibition of SNHG3 silencing on glioma cell progression. Besides, miR-384 inhibitor attenuated SNHG3 silencing induced inhibition on HDGF mRNA and protein expression in A172 and SHG44 cells. LncRNA SNHG3 promotes cell proliferation, migration, and invasion in glioma by enhancing HDGF expression via miR-384 sponging, representing the promising targets for the development of novel therapeutic strategies.

## 1 Introduction

Glioma, a malignant primary brain tumor derived from the central nervous system, has a high mortality rate and has threatened the well-being of millions of patients [1,2]. Currently, various advanced intervention strategies, such as surgical resection, chemotherapy, and local irradiation, have been achieved; however, 5 year survival rate of glioma is still less than 5% [3,4,5]. Therefore, investigation of the biological mechanism and development of novel therapeutic strategies are needed urgently.

Long non-coding RNAs (lncRNAs) are a subtype of RNAs comprising more than 200 nucleotides. They are essential modulators in cell infiltration, metabolism, differentiation, and metastasis by targeting the microRNAs (miRNAs) and participating in epigenetic transcriptional regulation [6,7,8]. Many lncRNAs have been identified as diagnostic or prognostic biomarkers in multiple cancers, such as nasopharyngeal carcinoma, hepatocellular carcinoma, and glioma [9,10,11]. Small nucleolar RNA host gene 3 (SNHG3), which is located on 1q35.3, is a novel lncRNA and closely related to tumor development [12]. For example, overexpression of SNHG3 promoted the invasive and migratory potentials by targeting miRNA-151a-3p to enhance RAB22A expression in osteosarcoma [13]. Likewise, SNHG3 served as an oncogene to enhance glioma malignant progression by KLF2 and p21 silencing [14]. Liu et al. discovered that SNHG3 was upregulated in lung adenocarcinoma tissues and abundance of SNHG3 promoted cell cycle and proliferation and inhibited apoptosis of lung adenocarcinoma [15]. However, the molecular mechanism in glioma requires an in-depth investigation.

MiRNAs are small chain RNAs with limited protein coding ability and they typically participate in many cellular physiological and pathological processes by altering gene expression at the post-transcription level, leading to mRNA degradation and protein translation suppression [16,17,18]. Aberrant expression of miRNAs is one of the leading causes of tumorigenesis and metastasis [19]. Upregulation of miR-384 suppressed tumor development, while deficiency of miR-384 expedited growth and metastasis by targeting SLBP in osteosarcoma MG63 cells [20]. Similarly, miR-384 induced cell apoptosis and autophagy through downregulation of collagen α-1(X) chain gene in non-small cell lung cancer [21]. Besides, miR-384 was found to repress proliferation, metastasis, and lipogenesis by targeting pleiotrophin in hepatitis B virus induced hepatocellular carcinoma [22]. However, whether miR-384 is involved in glioma development is largely obscure.

In the present study, we suggested that SNHG3 exerts oncogenic function by modulating miR-384/hepatoma-derived growth factor (HDGF) axis. The expression of SNHG3 and HDGF was upregulated while miR-384 was downregulated in tumor tissues, compared with normal tissues. Luciferase reporter system further identified the interaction between miR-384 and SNHG3 or HDGF. Moreover, miR-384 inhibitor inversed SNHG3 silencing mediated inhibition on HDGF mRNA and protein expression in A172 and SHG44 cells, implicating that SNHG3 could regulate HDGF expression by competing with miR-384.

## 2 Materials and methods

### 2.1 Patient tissues

A total of 42 glioma patients were recruited from Zhangzhou Affiliated Hospital of Fujian Medical University. All the patients had not received chemotherapy, radiotherapy, or other combined treatments prior to surgery. The tumor tissues and the corresponding normal tissues were obtained from the recruited volunteers by surgical resection and stored at −80°C immediately.


**Informed consent:** Informed consent has been obtained from all individuals included in this study.
**Ethical approval:** The research related to human use has been complied with all the relevant national regulations, institutional policies and in accordance with the tenets of the Helsinki Declaration and has been approved by the Ethics Committee of Zhangzhou Affiliated Hospital of Fujian Medical University.

### 2.2 Quantitative reverse transcription-polymerase chain reaction (qRT-PCR)

Glioma tissues and cells were incubated with TRIzol reagent (Invitrogen) to extract total RNA. Then, a NanoDrop ND-1000 Spectrophotometer (NanoDrop, Wilmington, MA, USA) was used to quantify RNA, and the purity was measured using the A260/280 ratio. Reverse transcription assay (5 µg RNA) was performed to synthesize cDNA using All-in-One™ First-Strand cDNA Synthesis Kit (FulenGen, Guangzhou, China). Briefly, the reverse transcription was conducted in a 10 µL reaction mixture, including 100 ng polyadenylated RNA, 2 µL of 5× PrimeScript Buffer, 0.5 µL of PrimeScript RT Enzyme Mix I, 1 µL of RT primer mixture, and RNase-free water. Then, the reaction mixture was incubated at 50°C for 15 min and 85°C for 5 s. Subsequently, qPCR was conducted using SYBR green (Applied Biosystems, Foster City, CA, USA) in a 15 µL final volume containing 1.5 µL of template cDNA mixed with 7.5 µL of 2× SYBR Green PCR master mix and 3 µL of each forward and reverse primers, according to the standard procedure. The relative expression was calculated using the 2^−ΔΔCt^ method. The amplification parameters were as follows: denaturation at 95°C for 10 min, followed by 40 cycles of denaturation at 95°C for 30 s, annealing at 60°C for 30 s, and extension at 72°C for 1 min. The primers for SNHG3, miR-384, and HDGF were as follows: SNHG3 forward 5′-TTCCGGGCGTTACTTAAGG-3′, reverse 5′-GGTCAAGAACAAGCACACCAA-3′; miR-384 forward 5′-TGTTAAATCAGGAATTTTAA-3′, reverse 5′-TGTTACAGGCATTATGAA-3′; HDGF forward 5′-GATCCCCGGCAGAAGGAGTACAAATTCAAGAGATTTGTACTCCTTCTGCCGGTTTTTTGGAAA-3′, reverse 5′-AGCTTTTCCAAAAAACCGGCAGAAGGAGTACAAATGTGTTGAATTTGTACTCCTTCTGCCGGG-3′.

### 2.3 Cell transfection

Glioma cell lines A172 and SHG44 and human astrocyte cell line NHA were purchased from iCell Bioscience Inc. (Shanghai, China). The cells were incubated in Dulbecco’s modified Eagle’s medium (DMEM; Gibco, Grand Island, NY, USA) comprising 10% fetal bovine serum (FBS) and 0.05% penicillin/streptomycin (Invitrogen, CA, USA) at 37^o^C in 5% CO_2_ incubator.

SNHG3 and HDGF overexpression vectors were obtained by cloning the sequences of SNHG3 and HDGF (GenePharma, Shanghai, China) into pcDNA3.1 (Invitrogen, Carlsbad, CA, USA), termed as pcDNA-SNHG3 and pcDNA-HDGF. Before transfection, A172 and SHG44 cells (2 × 10^5^ cells per well) were seeded into a 12-well plate. After incubation for 24 h, 0.2 µg of SNHG3 or HDGF overexpression vector (pcDNA-SNHG3 or pcDNA-HDGF) was transfected into A172 and SHG44 cells using 0.5 µL of Lipofectamine 2000 reagent (Invitrogen). Oligonucleotides including small interfering RNAs (siRNAs) targeting SNHG3 (si-SNHG3), HDGF (si-HDGF), and siRNA control (si-control) were synthesized by GenePharma, whereas miR-384 mimics, miR-384 inhibitor, inhibitor negative control (inhibitor-NC), and miRNA negative control (miR-NC) were purchased from RIBOBIO (Guangzhou, China). A172 and SHG44 cells were transfected with 0.5 µg of the aforementioned oligonucleotides using 0.6 µL of Lipofectamine 2000 (Invitrogen). After transfection for 48 h, the transfected cells were used for subsequent experiments.

### 2.4 Luciferase reporter assay

StarBase v2.0 and TargetScan predicted the potential binding sites between miR-384 and SNHG3 or HDGF. SNHG3 sequence or HDGF 3′-UTR sequence containing wild-type or mutant-type putative binding sites to miR-384 was cloned into pmirGLO vector (Promega, Madison, Wisconsin, USA), termed as SNHG3 WT or SNHG3 MUT, and HDGF 3′-UTR WT or HDGF 3′-UTR MUT. DNA sequencing was used to detect the reporters. Then, 400 ng of the constructed plasmids, 50 ng of Renilla luciferase reporter plasmid (pRL-TK), and 50 nM of miR-384 mimics or miR-NC were transfected into A172 and SHG44 cells using Lipofectamine 2000 (Invitrogen). After incubation for 48 h, a microplate reader (Biotek Instruments) and dual luciferase reporter assay kit (Promega) were used to detect the relative luciferase activity. Renilla luciferase activity was used to normalize the firefly luciferase activity.

### 2.5 CCK8 assay

For the CCK8 assay, A172 and SHG44 cells were seeded on a 96-well plate at a density of 5,000 cells/well and continuously cultured for 24, 48, and 72 h. After reacting with 10 µL of CCK8 reagent (Beyotime, Shanghai, China) for 2 h, the optical density value at 450 nm was measured by a microplate reader (Bio-Rad, Hercules, CA, USA).

### 2.6 Apoptosis assay

The fluorescein isothiocyanate (FITC) Annexin V-propidium iodide (PI) Kit (Beyotime) was used to detect cell apoptosis. A172 and SHG44 cells were harvested and co-stained with Annexin V-FITC/PI for 30 min at 48 h post-transfection. A172 and SHG44 cells were harvested and washed twice using ice-cold phosphate-buffered saline. Cells were resuspended using Annexin V binding buffer for the concentration of 0.25–1  ×  10^7^ cells/mL. Then, cells were incubated with 5 µL of FITC-Annexin V and 5 µL of PI for 15 min at room temperature in the dark. Stained cells were added with 400 µL of Annexin V binding buffer in each tube, and flow cytometry was used to analyze apoptosis.

**Table 1:** Relationship between SNHG3 expression and clinicopathologic features of glioma patients

	Characteristics (*n* = 42)	SNHG3 expression		*P* value^a^
		Low (*n* = 21)	High (*n* = 21)	
Gender				0.5366
Female	20	9	11	
Male	22	12	10	
Age (years)				0.5329
≤45	18	10	8	
>45	24	11	13	
Tumor location				0.4945
Supratentorial	30	14	16	
Infratentorial	12	7	5	
Tumor size				0.0300*
≤3 cm	19	13	6	
>3 cm	23	8	15	
WHO grade				0.0278*
I + II	25	16	9	
III + IV	17	5	12	

WHO: World Health Organization. *P < 0.05. ^a^Chi-square test.

### 2.7 Transwell assay

A172 and SHG44 cells (2 × 10^4^ cells per well) were plated into a 6-well plate and starved for 24 h. For transwell assay, A172 and SHG44 cells resuspended in serum-free DMEM (Gibco) were seeded on the upper chamber pre-coated with Matrigel (Becton Dickinson, Franklin Lakes, NJ, USA) for invasion assay (without Matrigel coating for migration assay). Meanwhile, the lower chamber containing the medium supplemented with 10% FBS (Gibco) was regarded as a chemoattractant. After incubation for 48 h, the non-invasive and non-migrated cells were scraped, while the migrated and invaded cells at the lower chamber were fixed with methanol and stained with 0.1% crystal violet (Sigma, St. Louis, MO, USA) for 10 min and counted using a microscope.

### 2.8 Statistical analysis

Data were presented as mean ± standard deviation. Statistical analysis was carried out by SPSS 13.0 software (Chicago, IL, USA) and GraphPad Prism 7 (GraphPad Inc., San Diego, CA, USA). The correlation of miR-384 and SNHG3 or HDGF was determined by Pearson’s correlation coefficient analysis. A *P*-value of <0.05 was considered statistically significant.

## 3 Results

### 3.1 Upregulation of SNHG3 in glioma

**Figure 1:**
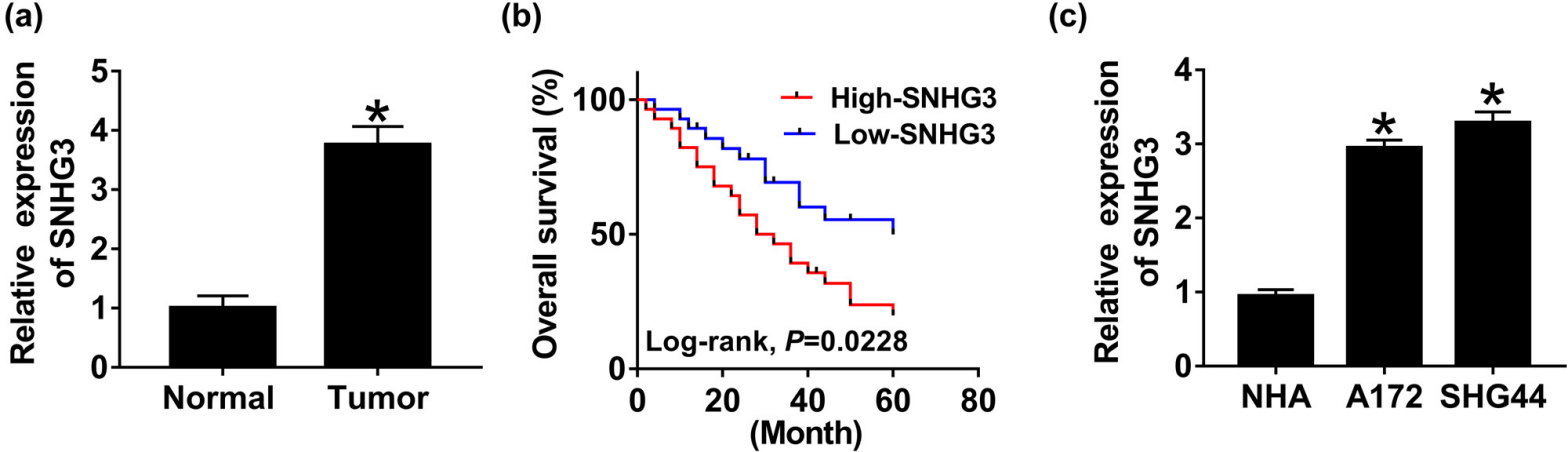
SNHG3 was upregulated in glioma tumors and cell lines. (a) SNHG3 expression in tumor tissues compared with the adjacent normal tissues measured by qRT-PCR. (b) The survival rate of glioma patients with different SNHG3 levels in 60 days. *P = 0.0228. (c) SNHG3 expression in glioma cell lines A172 and SHG44, compared with human astrocyte cell line NHA. *P < 0.05.

The expression of SNHG3 in 42 pairs of glioma tumors and normal tissues was determined using qRT-PCR to elucidate the role of SNHG3 in glioma development. As illustrated in Figure 1a, an obvious upregulation of SNHG3 was observed in tumors, compared with the corresponding normal tissues. Consistently, SNHG3 expression was upregulated dramatically in glioma cell lines A172 and SHG44 in comparison with human astrocyte cell line NHA (Figure 1c). Noticeably, high level of SNHG3 resulted in low survival rate, whereas low level of SNHG3 resulted in high survival rate within 60 days in glioma patients (Figure 1b). Collectively, we suggested that SNHG3 plays an oncogenic role in glioma.

### 3.2 SNHG3 knockdown suppresses cell progression and induces apoptosis in glioma

**Figure 2:**
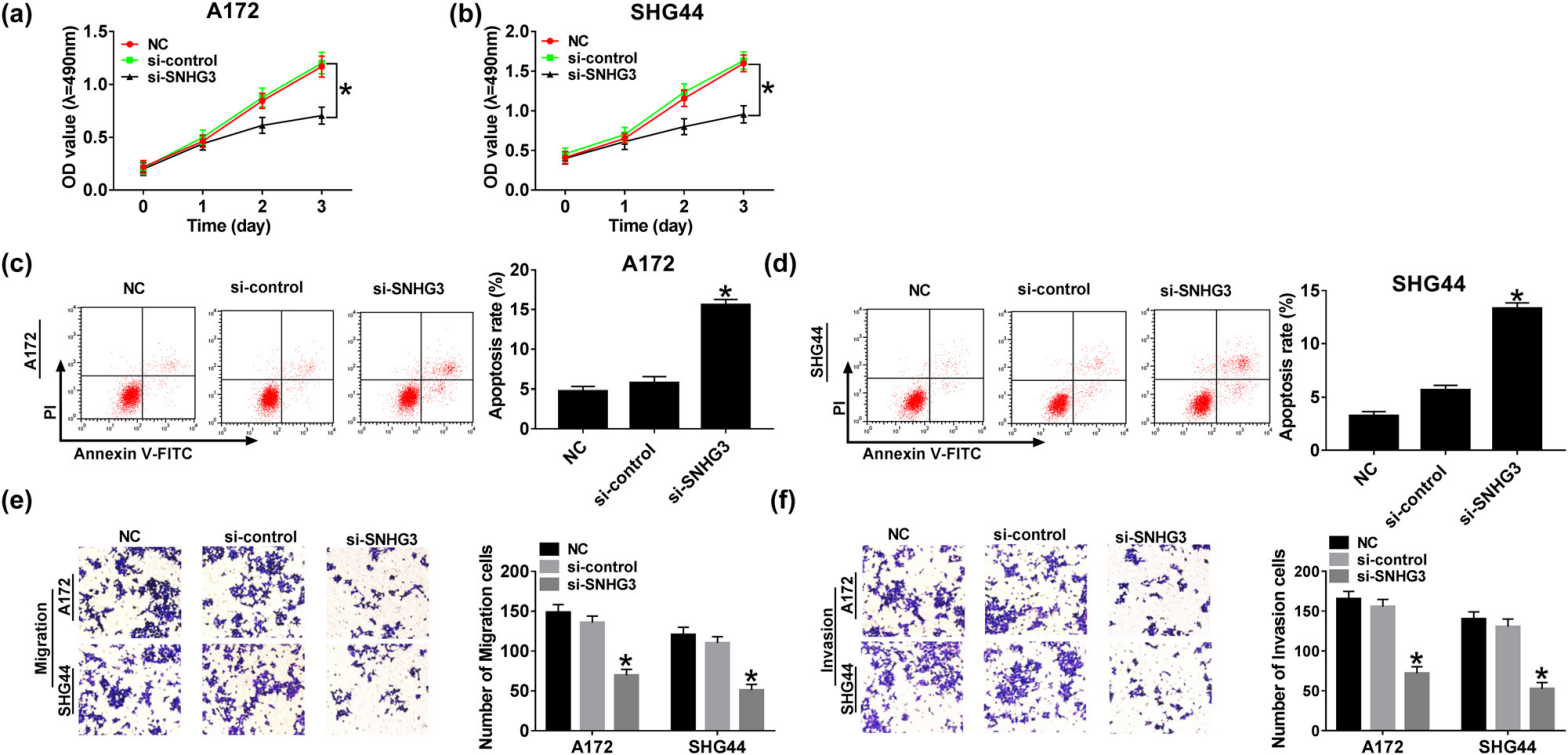
SNHG3 knockdown hindered proliferation, migration, and invasion and induced apoptosis in glioma. A172 and SHG44 cells were transfected with si-SNHG3 and si-control. Cell viability of A172 (a) and SHG44 cells (b) at different times (1, 2, and 3 days) post-transfection. Cell apoptotic rate of A172 (c) and SHG44 cells (d) at 48 h post-transfection. Cell migration (e) and invasion ability (f) of A172 and SHG44 cells at 48 h post-transfection determined by transwell assay. *P < 0.05.

Identification of the function of SNHG3 in glioma cell progression was evaluated by CCK8, flow cytometry, and transwell assay. Initially, A172 and SHG44 cells were transfected with si-SNHG3 and si-control for the following biological investigation. We noticed that cell viability was repressed significantly in A172 and SHG44 cells after SNHG3 silencing, compared with the si-control group (Figure 2a and b). Consistent with CCK8 results, transwell assay exhibited that migration and invasion ability were suppressed in glioma cells transfected with si-SNHG3 while remaining unchanged in the si-control group (Figure 2e and f). As expected, SNHG3 knockdown induced apoptosis largely in A172 and SHG44 cells (Figure 2c and d). Those findings represented that SNHG3 could accelerate progression and suppress apoptosis in glioma.

### 3.3 HDGF knockdown inhibits cell progression and enhances apoptosis in glioma

**Figure 3:**
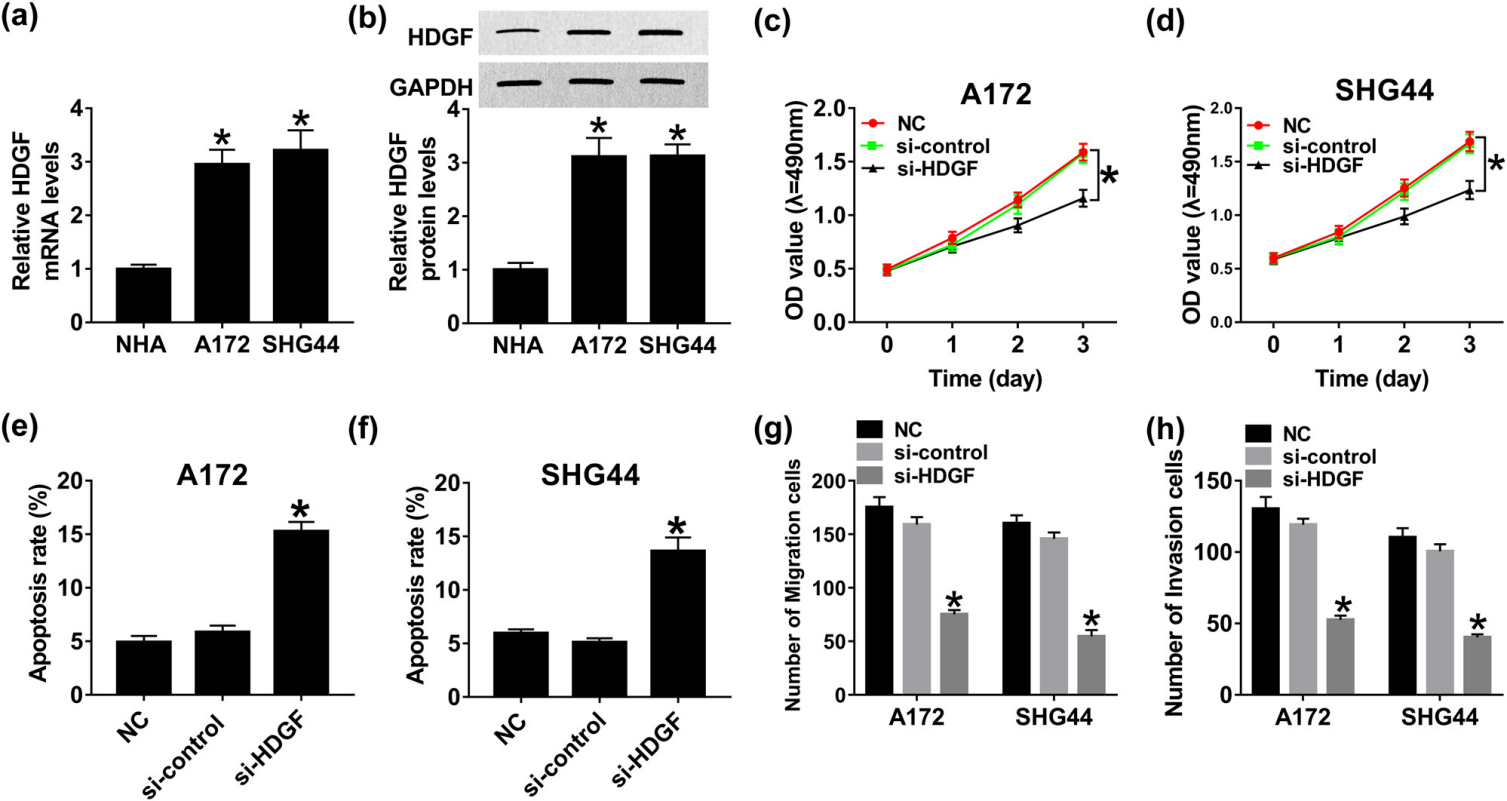
HDGF knockdown inhibited proliferation, migration, and invasion and enhanced apoptosis in glioma. A172 and SHG44 cells were transfected with si-HDGF and si-control. The expression of HDGF mRNA (a) and protein (b) in A172 and SHG44 cells at 48 h post-transfection was examined by qRT-PCR and western blot, respectively. Cell viability of A172 (c) and SHG44 cells (d) at different times (1, 2, and 3 days) post-transfection. Cell apoptotic rate of A172 (e) and SHG44 cells (f) at 48 h post-transfection. Cell migration (g) and invasion ability (h) of A172 and SHG44 cells at 48 h post-transfection. *P < 0.05.

Subsequently, we transfected si-HDGF and si-control in A172 and SHG44 cells to explore the influence of HDGF on glioma cell proliferation, apoptosis, migration, and invasion. As displayed in Figure 3a and b, distinct improvement of HDGF mRNA and protein expression in A172 and SHG44 cells was discovered after si-HDGF transfection in comparison with si-control transfection, representing that the transfection efficiency is extremely high. CCK8 results indicated that downregulation of HDGF remarkably inhibited proliferation ability of A172 and SHG44 cells (Figure 3c and d). Likewise, the number of migration and invasion cells was reduced in glioma cells after HDGF silencing (Figure 3g and h). On the contrary, the apoptotic rate was elevated in A172 and SHG44 cells transfected with si-HDGF, compared with si-control (Figure 3e and f). Therefore, we concluded that HDGF functions as an oncogene to accelerate cell growth as well as to suppress apoptosis in glioma.

### 3.4 Restoration of HDGF abrogates the inhibition of SNHG3 silencing on proliferation, migration, and invasion in glioma

**Figure 4:**
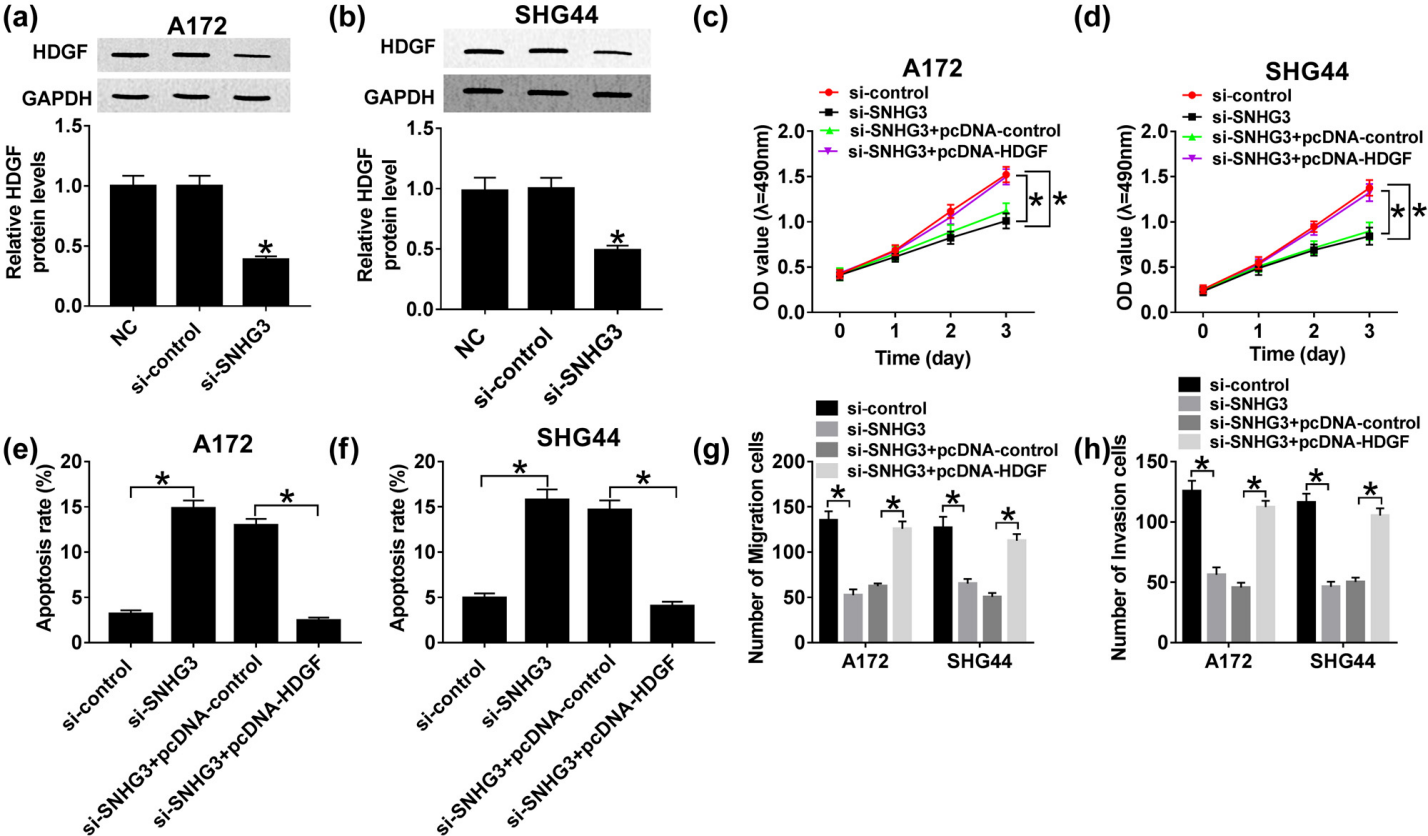
HDGF attenuated SNHG3 silencing induced inhibition on cell progression in glioma. A172 and SHG44 cells were transfected with si-SNHG3 + pcDNA-HDGF, si-SNHG3 + pcDNA-control, si-SNHG3, and si-control. The expression of HDGF protein in transfected A172 (a) and SHG44 cells (b). Cell viability of transfected A172 (c) and SHG44 cells (d) cells at different times (1, 2, and 3 days) post-transfection. Cell apoptotic rate of A172 (e) and SHG44 cells (f) cells at 48 h post-transfection. Cell migration (g) and invasion ability (h) of A172 and SHG44 cells at 48 h post-transfection. *P < 0.05.

To clarify the regulatory effects of SNHG3/HDGF axis on glioma cell progression, A172 and SHG44 cells were transfected with si-SNHG3, si-SNHG3 + pcDNA-HDGF, si-SNHG3 + pcDNA-control, and si-control. Through western blot analysis, we discovered that HDGF protein expression was decreased distinctly in A172 and SHG44 cells after SNHG3 knockdown, indicating that SNHG3 might modulate HDGF positively (Figure 4a and b). Moreover, rescue experiments implicated that restoration of HDGF abrogated SNHG3 silencing induced suppressive effect on glioma cell proliferation (Figure 4c and d). Similarly, HDGF deficiency reduced the number of migratory and invasive cells; however, the abundance of HDGF showed the opposite trend (Figure 4g and h). Interestingly, HDGF rescued SNHG3 silencing mediated promotion on apoptosis in glioma (Figure 4e and f). Altogether, SNHG3 promotes glioma cell development by regulating HDGF.

### 3.5 SNHG3 is a sponger of miR-384

**Figure 5:**
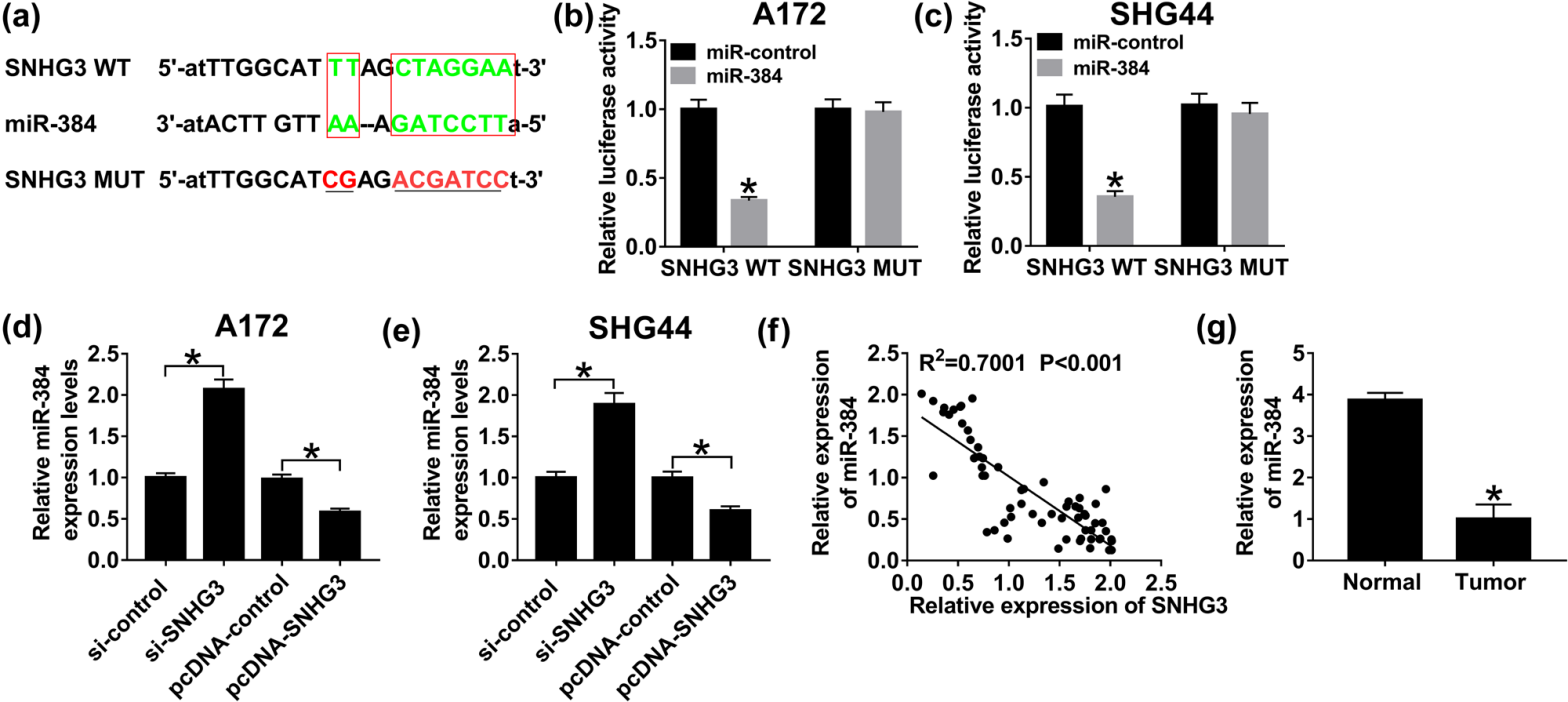
SNHG3 directly interacts with miR-384. (a) The putative binding sites of SNHG3 and miR-384 searched by StarBase v2.0. Luciferase activity of A172 (b) and SHG44 cells (c) co-transfected with SNHG3 WT or SNHG3 MUT and miR-384 mimics or miR-NC. The expression of miR-384 in A172 (d) and SHG44 cells (e) transfected with pcDNA-SNHG3, pcDNA-NC, si-SNHG3, and si-control. (f) The correlation between SNHG3 and miR-384 analyzed by Pearson’s correlation coefficient (*R* ^2^ = 0.7001, *P* < 0.001). (g) The expression of miR-384 in glioma tissues and the adjacent normal tissues. *P < 0.05.

Increasing evidence has identified that lncRNA acts as a competing endogenous RNA (ceRNA) to modulate cell behavior by competing with its miRNA; thus, we searched the online database StarBase v2.0 and found that miR-384 has the potential to bind to SNHG3 (Figure 5a). To certify the prediction, wild-type SNHG3 (SNHG3 WT) and mutant-type (SNHG3 MUT) vectors were constructed and subsequently co-transfected with miR-384 mimics and miR-NC in A172 and SHG44 cells. Reduction of luciferase activity had appeared in both A172 and SHG44 cells co-transfected with SNHG3 WT and miR-384 mimics, confirming the interaction between SNHG3 and miR-384 (Figure 5b and c). In addition, sufficiency of SNHG3 reduced miR-384, whereas deficiency of SNHG3 elevated miR-384 expression in glioma cells (Figure 5d and e). Besides, miR-384 expression was downregulated in tumor tissues, compared with normal tissues (Figure 5g). SNHG3 was inversely correlated with miR-384 (*R* ^2^ = 0.7001, *P* < 0.001) (Figure 5f). Taking together, SNHG3 serves as a sponger of miR-384.

### 3.6 HDGF is a target gene of miR-384

**Figure 6:**
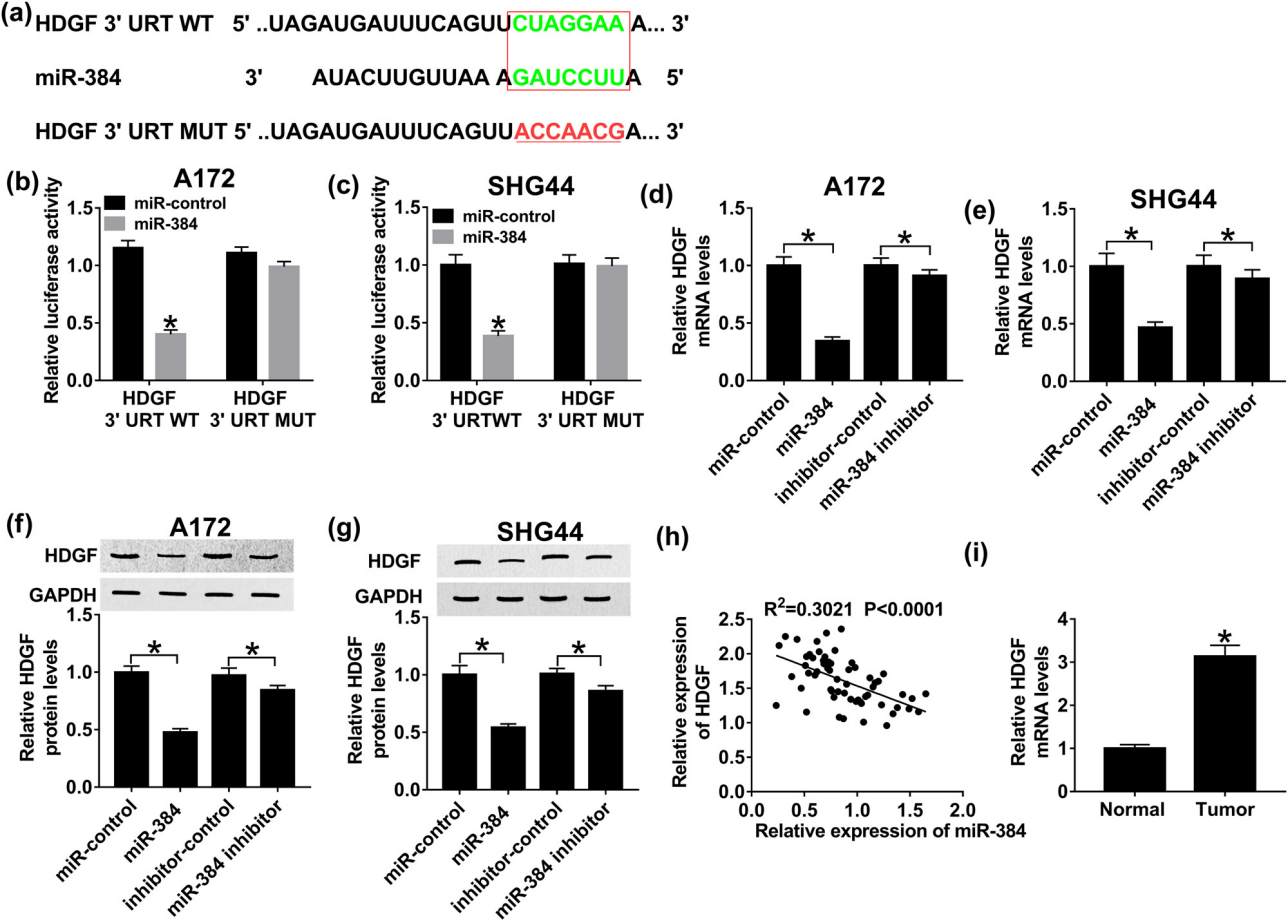
The interaction between HDGF and miR-384. (a) The putative binding sites of HDGF and miR-384 were predicted by TargetScan. Luciferase activity of A172 (b) and SHG44 cells (c) co-transfected with HDGF 3′-UTR WT or HDGF 3′-UTR MUT and miR-384 mimics or miR-NC. HDGF mRNA expression in A172 (d) and SHG44 cells (e) transfected with miR-384 mimics, miR-384 inhibitor, inhibitor-NC, and miR-NC. HDGF protein expression in A172 (f) and SHG44 cells (g) transfected with miR-384 mimics, miR-384 inhibitor, inhibitor-NC, and miR-NC. (h) The correlation between HDGF and miR-384 (*R* ^2^ = 0.3021, *P* < 0.0001). (i) HDGF expression in tumor tissues, compared with normal tissues. *P < 0.05.

Based on TargetScan (http://www.targetscan.org) prediction, miR-384 comprises the potential binding sites of 3′-UTR HDGF (Figure 6a). Then, luciferase reporter system was constructed by co-transfecting HDGF 3′-UTR WT or HDGF 3′-UTR MUT and miR-384 mimics or miR-NC in A172 and SHG44 cells to determine the interaction between HDGF and miR-384. As illustrated in Figure 6b and c, luciferase activity decreased apparently in HDGF 3′-UTR WT and miR-384 mimic co-transfection cells. By contrast, luciferase activity remained unchanged in the HDGF 3′-UTR MUT transfection group. Moreover, HDGF mRNA levels were repressed by miR-384 mimics and boosted by miR-384 inhibitor in glioma (Figure 6d and e). Meanwhile, upregulation of miR-384 hindered HDGF protein expression; however, downregulation of miR-384 showed the opposite effect (Figure 6f and g). In addition, HDGF expression was upregulated in tumor tissues, compared with normal tissues (Figure 6i). By calculation, HDGF was correlated with miR-384 negatively (*R* ^2^ = 0.3021, *P* < 0.0001) (Figure 6h). These data clarified that HDGF is a target gene of miR-384.

### 3.7 SNHG3 modulates HDGF expression by sponging miR-384 in glioma

**Figure 7:**
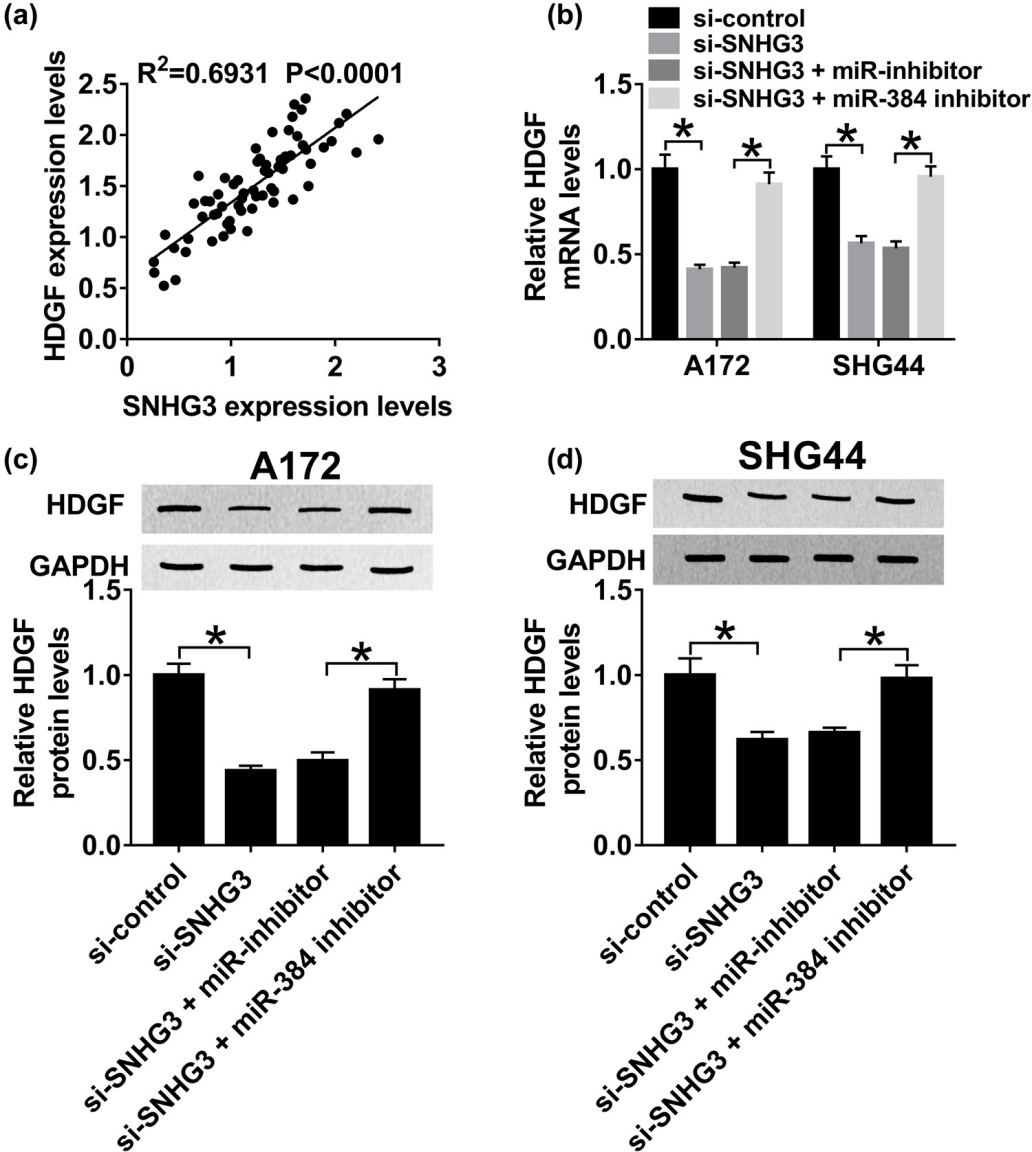
SNHG3 modulates HDGF expression by sponging miR-384 in glioma. A172 and SHG44 cells were transfected with si-SNHG3, si-SNHG3 + miR-384 inhibitor, si-SNHG3 + miR-NC, and si-control. (a) The correlation between HDGF and SNHG3 (*R* ^2^ = 0.6931, *P* < 0.0001). (b) HDGF mRNA expression in transfected A172 and SHG44 cells. HDGF protein expression in transfected A172 (c) and SHG44 cells (d). *P < 0.05.

The regulatory network of SNHG3/miR-384/HDGF axis in glioma progression was validated by qRT-PCR and western blot assay. First, we noticed that SNHG3 was positively correlated with HDGF (*R* ^2^ = 0.6931, *P* < 0.0001) (Figure 7a). More importantly, HDGF mRNA expression was decreased after SNHG3 silencing and increased by miR-384 inhibitor (Figure 7b). Consistently, miR-384 inhibitor restored SNHG3 silencing mediated inhibition on HDGF protein expression in A172 and SHG44 cells (Figure 7c and d). These results elucidated that SNHG3 upregulates HDGF expression by sponging miR-384 in glioma.

## 4 Discussion

Accumulating evidence has elucidated that lncRNA could modulate cell growth and gene expression by interacting with miRNA as a ceRNA [23]. SNHG3 has been regarded as a prognostic factor in multiple cancer types since its expression level is associated with portal vein tumor thrombus, tumor size, and recurrence [24,25]. For instance, upregulation of SNHG3 indicates poor survival since SNHG3 could accelerate cell growth by sponging miR-196a-5p in osteosarcoma patients [26]. In addition, alteration of SNHG3 expression is associated with the onset of many cancers, for example, SNHG3 functioned as ceRNA by sponging miR-182-5p to release c-Myc and promote malignant development in colorectal cancer [27]. Similarly, SNHG3 repressed the degradation of CD151 by competing with miR-128, contributing to the epithelial–mesenchymal transition (EMT) and sorafenib resistance in hepatocellular carcinoma [28]. Therefore, it is of great significance to illuminate the role of SNHG3 in glioma.

Numerous research studies have demonstrated that miRNAs regulate the downstream gene and protein expression by base-pairing of 3′-UTR of mRNA to control cell behavior in cancers [29,30,31]. Differentially, expression of miR-384 was diagnosed in multiple cancers, for instance, overexpression of miR-384 inhibited gastric cancer cell growth, migration, and invasion by interacting with metadherin [32,33]. In addition, miR-84 suppressed cell proliferation, migration, and metastasis in renal cell carcinoma and colorectal cancer by targeting and interacting with RAB23 and KRAS/CDC42 axis, respectively [34,35]. Promotion of miR-384 was indicated to inhibit cell growth and EMT by sponging lncRNA TUG1 in nasopharyngeal carcinoma [36]. Therefore, identification of the potential functional miRNA might present novel targets for glioma diagnosis.

In our study, we attempted to illuminate the biological mechanism of SNHG3 on glioma tumorigenesis, proliferation, migration, invasion, and apoptosis processes. It is apparent that SNHG3 and HDGF expression was upregulated while miR-384 was downregulated in tumor tissues. In addition, SNHG3 or HDGF knockdown significantly attenuated glioma cell progression; however, restoration of HDGF expression remarkably enhanced proliferation, migration, and invasion in glioma. The subsequent luciferase activity detection confirmed the interaction between miR-384 and SNHG3 or HDGF. Besides, Pearson’s correlation coefficient analysis revealed that miR-384 was correlated with SNHG3 or HDGF inversely. Essentially, miR-384 inhibitor abolished the suppression of SNHG3 silencing on HDGF mRNA and protein expression, validating that SNHG3 accelerates cell progression and induces apoptosis by upregulating HDGF expression via sponging miR-384 in glioma.

In conclusion, we clarified the regulatory effect of SNHG3/miR-384/HDGF axis on glioma cell proliferation, migration, invasion, and apoptosis. We discovered that SNHG3 acts as an oncogene in glioma to promote cell progression by upregulating HDGF expression through interaction with miR-384. Our research results might represent potential targeted therapy which is of great clinical significance.

## References

[1] Qiu XG, Chen YD, Yuan J, Zhang N, Lei T, Liu J, et al. Functional BCL-2 rs2279115 promoter noncoding variant contributes to glioma predisposition, especially in males. DNA Cell Biol. 2019;38(1):85–90. 10.1089/dna.2018.4318 30481055

[2] Sun D, Mu Y, Piao H. MicroRNA-153-3p enhances cell radiosensitivity by targeting BCL2 in human glioma. Biol Res. 2018;51(1):56. 10.1186/s40659-018-0203-6 PMC6288870 30537994

[3] Zhang Y, Sui R, Chen Y, Liang H, Shi J, Piao H. Long noncoding RNA MT1JP inhibits proliferation, invasion, and migration while promoting apoptosis of glioma cells through the activation of PTEN/Akt signaling pathway. J Cell Physiol. 2019;234:19553–64. 10.1002/jcp.28553 31066040

[4] Wang R, Bao HB, Du WZ, Chen XF, Liu HL, Han DY, et al. P68 RNA helicase promotes invasion of glioma cells through negatively regulating DUSP5. Cancer Sci. 2019;110(1):107–17. 10.1111/cas.13858 PMC6317933 30387548

[5] Wu HL, Fu XY, Cao WQ, Xiang WZ, Hou YJ, Ma JK, et al. Induction of apoptosis in human glioma cells by fucoxanthin via triggering of ROS-mediated oxidative damage and regulation of MAPKs and PI3K-AKT pathways. J Agric Food Chem. 2019;67(8):2212–9. 10.1021/acs.jafc.8b07126 30688446

[6] Li X, Zhang H, Wu X. Long noncoding RNA DLX6-AS1 accelerates the glioma carcinogenesis by competing endogenous sponging miR-197-5p to relieve E2F1. Gene. 2019;686:1–7. 10.1016/j.gene.2018.10.065 30366080

[7] Gao L, Wang X, Guo S, Xiao L, Liang C, Wang Z, et al. LncRNA HOTAIR functions as a competing endogenous RNA to upregulate SIRT1 by sponging miR-34a in diabetic cardiomyopathy. J Cell Physiol. 2019;234(4):4944–58. 10.1002/jcp.27296 30216438

[8] Li J, Li Y, Meng F, Fu L, Kong C. Knockdown of long non-coding RNA linc00511 suppresses proliferation and promotes apoptosis of bladder cancer cells via suppressing Wnt/beta-catenin signaling pathway. Biosci Rep. 2018;38(4):BSR20171701. 10.1042/BSR20171701 PMC6131201 30042171

[9] Gong X, Liao X, Huang M. LncRNA CASC7 inhibits the progression of glioma via regulating Wnt/beta-catenin signaling pathway. Pathol Res Pract. 2019;215(3):564–70. 10.1016/j.prp.2019.01.018 30661904

[10] Su H, Liu L, Zhang Y, Wang J, Zhao Y. Long noncoding RNA NPCCAT1 promotes nasopharyngeal carcinoma progression via upregulating YY1. Biochimie. 2019;157:184–94. 10.1016/j.biochi.2018.11.014 30481541

[11] Cui M, Chen M, Shen Z, Wang R, Fang X, Song B. LncRNA-UCA1 modulates progression of colon cancer through regulating the miR-28-5p/HOXB3 axis. J Cell Biochem. 2019;120:6926–36. 10.1002/jcb.27630 30652355

[12] Li N, Zhan X, Zhan X. The lncRNA SNHG3 regulates energy metabolism of ovarian cancer by an analysis of mitochondrial proteomes. Gynecol Oncol. 2018;150(2):343–54. 10.1016/j.ygyno.2018.06.013 29921511

[13] Zheng S, Jiang F, Ge D, Tang J, Chen H, Yang J, et al. LncRNA SNHG3/miRNA-151a-3p/RAB22A axis regulates invasion and migration of osteosarcoma. Biomed Pharmacother. 2019;112:108695. 10.1016/j.biopha.2019.108695 30797154

[14] Fei F, He Y, He S, He Z, Wang Y, Wu G, et al. LncRNA SNHG3 enhances the malignant progress of glioma through silencing KLF2 and p21. Biosci Rep. 2018;38(5):BSR20180420. 10.1042/BSR20180420 PMC6127675 30042166

[15] Liu L, Ni J, He X. Upregulation of the long noncoding RNA SNHG3 promotes lung adenocarcinoma proliferation. Dis Markers. 2018;2018:5736716. 10.1155/2018/5736716 PMC6081568 30154938

[16] Li J, Li Q, Lin L, Wang R, Chen L, Du W, et al. Targeting the Notch1 oncogene by miR-139-5p inhibits glioma metastasis and epithelial-mesenchymal transition (EMT). BMC Neurol. 2018;18(1):133. 10.1186/s12883-018-1139-8 PMC6117922 30170559

[17] Huang S, Zheng S, Huang S, Cheng H, Lin Y, Wen Y, et al. Flot2 targeted by miR-449 acts as a prognostic biomarker in glioma. Artif Cells Nanomed Biotechnol. 2019;47(1):250–5. 10.1080/21691401.2018.1549062 30663389

[18] Zhu Y, Gu J, Li Y, Peng C, Shi M, Wang X, et al. MiR-17-5p enhances pancreatic cancer proliferation by altering cell cycle profiles via disruption of RBL2/E2F4-repressing complexes. Cancer Lett. 2018;412:59–68. 10.1016/j.canlet.2017.09.044 28987387

[19] Yang R, Xing L, Zheng X, Sun Y, Wang X, Chen J. The circRNA circAGFG1 acts as a sponge of miR-195-5p to promote triple-negative breast cancer progression through regulating CCNE1 expression. Mol Cancer. 2019;18(1):4. 10.1186/s12943-018-0933-7 PMC6325825 30621700

[20] Wang Y, Huang H, Li Y. Knocking down miR-384 promotes growth and metastasis of osteosarcoma MG63 cells by targeting SLBP. Artif Cells Nanomed Biotechnol. 2019;47(1):1458–65. 10.1080/21691401.2019.1601099 31007083

[21] Guo Q, Zheng M, Xu Y, Wang N, Zhao W. MiR-384 induces apoptosis and autophagy of non-small cell lung cancer cells through the negative regulation of Collagen alpha-1(X) chain gene. Biosci Rep. 2019;39(2):BSR20181523. 10.1042/BSR20181523 PMC6356039 30442874

[22] Bai PS, Xia N, Sun H, Kong Y. Pleiotrophin, a target of miR-384, promotes proliferation, metastasis and lipogenesis in HBV-related hepatocellular carcinoma. J Cell Mol Med. 2017;21(11):3023–43. 10.1111/jcmm.13213 PMC5661149 28557334

[23] Zhao X, Liu Y, Li Z, Zheng S, Wang Z, Li W, et al. Linc00511 acts as a competing endogenous RNA to regulate VEGFA expression through sponging hsa-miR-29b-3p in pancreatic ductal adenocarcinoma. J Cell Mol Med. 2018;22(1):655–67. 10.1111/jcmm.13351 PMC5742682 28984028

[24] Zhang T, Cao C, Wu D, Liu L. SNHG3 correlates with malignant status and poor prognosis in hepatocellular carcinoma. Tumour Biol. 2016;37(2):2379–85. 10.1007/s13277-015-4052-4 26373735

[25] Hong L, Chen W, Wu D, Wang Y. Upregulation of SNHG3 expression associated with poor prognosis and enhances malignant progression of ovarian cancer. Cancer Biomark. 2018;22(3):367–74. 10.3233/CBM-170710 PMC13078481 29758922

[26] Chen J, Wu Z, Zhang Y. LncRNA SNHG3 promotes cell growth by sponging miR-196a-5p and indicates the poor survival in osteosarcoma. Int J Immunopathol Pharmacol. 2019;33:2058738418820743. 10.1177/2058738418820743 PMC6329016 30791797

[27] Huang W, Tian Y, Dong S, Cha Y, Li J, Guo X, et al. The long non-coding RNA SNHG3 functions as a competing endogenous RNA to promote malignant development of colorectal cancer. Oncol Rep. 2017;38(3):1402–10. 10.3892/or.2017.5837 PMC5549033 28731158

[28] Zhang PF, Wang F, Wu J, Wu Y, Huang W, Liu D, et al. LncRNA SNHG3 induces EMT and sorafenib resistance by modulating the miR-128/CD151 pathway in hepatocellular carcinoma. J Cell Physiol. 2019;234(3):2788–94. 10.1002/jcp.27095 30132868

[29] Zhang X, Yu J, Zhao C, Ren H, Yuan Z, Zhang B, et al. MiR-181b-5p modulates chemosensitivity of glioma cells to temozolomide by targeting Bcl-2. Biomed Pharmacother. 2019;109:2192–202. 10.1016/j.biopha.2018.11.074 30551476

[30] Zhu M, Wei C, Lin J, Dong S, Gao D, Chen J, et al. UHRF1 is regulated by miR-124-3p and promotes cell proliferation in intrahepatic cholangiocarcinoma. J Cell Physiol. 2019;234(11):19875–85. 10.1002/jcp.28586 30989656

[31] Zhou Y, Deng J, Chu X, Zhao Y, Guo Y. Role of post-transcriptional control of calpain by miR-124-3p in the development of Alzheimer’s disease. J Alzheimers Dis. 2019;67(2):571–81. 10.3233/JAD-181053 30584150

[32] Wang F. miR-384 targets metadherin gene to suppress growth, migration, and invasion of gastric cancer cells. J Int Med Res. 2019;47(2):926–35. 10.1177/0300060518817171 PMC6381512 30614349

[33] Lai YY, Shen F, Cai WS, Chen JW, Feng JH, Cao J, et al. MiR-384 regulated IRS1 expression and suppressed cell proliferation of human hepatocellular carcinoma. Tumour Biol. 2016;37(10):14165–71. 10.1007/s13277-016-5233-5 27542674

[34] Wang YX, Chen YR, Liu SS, Ye YP, Jiao HL, Wang SY, et al. MiR-384 inhibits human colorectal cancer metastasis by targeting KRAS and CDC42. Oncotarget. 2016;7(51):84826–38. 10.18632/oncotarget.12704 PMC5356701 27769041

[35] Yan L, Wu K, Du F, Yin X, Guan H. miR-384 suppressed renal cell carcinoma cell proliferation and migration through targeting RAB23. J Cell Biochem. 2019;120(2):1420–6. 10.1002/jcb.27180 30390327

[36] Qian W, Ren Z, Lu X. Knockdown of long non-coding RNA TUG1 suppresses nasopharyngeal carcinoma progression by inhibiting epithelial-mesenchymal transition (EMT) via the promotion of miR-384. Biochem Biophys Res Commun. 2019;509(1):56–63. 10.1016/j.bbrc.2018.12.011 30581000

